# Autobiographical Memory: A Clinical Perspective

**DOI:** 10.3389/fnbeh.2013.00194

**Published:** 2013-12-10

**Authors:** Nadja Urbanowitsch, Lina Gorenc, Christina J. Herold, Johannes Schröder

**Affiliations:** ^1^Section of Geriatric Psychiatry, University of Heidelberg, Heidelberg, Germany; ^2^Institute of Gerontology, University of Heidelberg, Heidelberg, Germany

**Keywords:** autobiographical memory, semantic memory, episodic memory, mild cognitive impairment, Alzheimer’s disease, chronic schizophrenia, hippocampus, multiple trace theory

## Abstract

Autobiographical memory (ABM) comprises memories of one’s own past that are characterized by a sense of subjective time and autonoetic awareness. Although ABM deficits are among the primary symptoms of patients with major psychiatric conditions such as mild cognitive impairment (MCI) and Alzheimer Disease (AD) or chronic schizophrenia large clinical studies are scarce. We therefore summarize and discuss the results of our clinical studies on ABM deficits in the respective conditions. In these studies ABM was assessed by using the same instrument – i.e., the Erweitertes Autobiographisches Gedächtnis Inventar (E-AGI) – thus allowing a direct comparison between diagnostic groups. Episodic ABM, especially the richness of details was impaired already in MCI and in beginning AD. Semantic memories were spared until moderate stages, indicating a dissociation between both memory systems. A recency effect was detectable in cognitively unimpaired subjects and vanished in patients with AD. A similar pattern of deficits was found in patients with chronic schizophrenia but not in patients with major depression. These ABM deficits were not accounted for by gender, or education level and did not apply for the physiological ageing process in otherwise healthy elderly. In conclusion, ABM deficits are frequently found in AD and chronic schizophrenia and primarily involve episodic rather than semantic memories. This dissociation corresponds to the multiple trace theory which hypothesized that these memory functions refer to distinct neuronal systems. The semi-structured interview E-AGI used to discern ABM changes provided a sufficient reliability measures, moreover potential effects of a number of important confounders could be falsified so far. These findings underline the relevance of ABM-assessments in clinical practice.

## Introduction

Autobiographical memory (ABM) refers to memories of an individual, which are characterized by a sense of subjective time and autonoetic awareness (Tulving, 1972, 2002) and entailed by feelings of emotional re-experience (Tulving, 1983; Tulving and Markowitsch, 1998; Markowitsch, 2003). Because of the interaction of episodic and semantic memory and the uniqueness to humans ABM is considered to be crucial for the continuity of the self and the development of personal identity, i.e., processes which are typically disturbed in patients with major psychiatric conditions such as Alzheimer’s disease (AD) or chronic schizophrenia (Conway and Pleydell-Pearce, 2000; Cuervo-Lombard et al., 2007; Berna et al., 2012; Seidl et al., 2011; Herold et al., 2013). As a part of the declarative memory, ABM comprises a semantic plus an episodic domain. While semantic ABM involves general facts from different life time periods, episodic ABM includes biographic events with a richness of details and a feeling of re-experiencing when recalled.

According to Ribot’s law (Ribot, 1881) remote memories are more resistant to brain damage than recent one. Ribot’s law stands in opposition to the recency effect that implies a better consolidation of recent memories than remote ones. Declarative mnestic deficits are among the core symptoms of AD and usually go along with anterograde memory impairment in the initial phases and loss of remote memory following Ribot’s gradient in the more advanced stages (Sagar et al., 1988; Dall’Ora et al., 1989; Kopelman, 1989; Greene and Hodges, 1996; Dorrego et al., 1999; Piolino et al., 2003; Hou et al., 2005; Leyhe et al., 2009). Two important theoretical approaches regarding the role of the hippocampus on ABM retrieval are the standard model of consolidation and the multiple trace theory (Squire and Alvarez, 1995; Nadel and Moscovitch, 1997). The first approach suggests that the function of the hippocampus in ABM is time-limited; hence, memories become gradually independent of the medial temporal lobe (MTL) in the course of time. In contrast, the multiple trace theory predicts that the recall process of the episodic autobiographical memories requires the hippocampal formation irrespective of how old the relevant memories are. The semantic memories, however, could be recalled independently of this structure and were subject to Ribot’s gradient. The majority of studies support the multiple trace theory (Conway et al., 1999; Piolino et al., 2004; Viard et al., 2007). There are also reports of spared personal-semantic memory but impaired personal episodic memories without a temporal gradient in patients with MTL lesions (Viskontas et al., 2000; Steinvorth et al., 2005; Noulhiane et al., 2008).

Autobiographical memory deficits are not specific to AD but were also described in mild cognitive impairment (MCI) and chronic schizophrenia. These changes do not only contribute to our understanding of the respective diseases but have the potential to facilitate clinical examination and diagnosis. However, the potential impact of important confounders, such as education, depressive mood, or the aging process as such needs to be addressed.

In the following we summarize and discuss findings from our studies on ABM deficits in MCI and AD, major depression, and chronic schizophrenia with reference to normal aging.

## Clinical Studies

### Methods

Methodological details of the five studies conducted by our group as well as the description of sample characteristics are summarized in Table 1.

**Table 1:** **Studies on ABM in major depression, MCI, AD, and chronic schizophrenia**.

Study	Psychometric instruments/neuropsychological assessment/MRI	Subjects				
		Sample size	Patients’ groups	Female/male	Age (years): mean (SD)	Education (years): mean (SD)
Ahlsdorf (2009)	Erweitertes Autobiographisches Gedächtnis Inventar (E-AGI), previous version	*N* = 120	Depression (*n* = 30)	21/9	68.8 (6.6)	11.8 (2.4)
	Mini mental state examination (MMSE)		MCI (*n* = 30)	15/15	70.2 (5.8)	12.3 (3.3)
	NEO five factor inventory (NEO-FFI)		AD (*n* = 30)	18/12	74.4 (6.7)	11.0 (2.7)
	Beck depression inventar (BDI)		Healthy controls (*n* = 30)	19/11	66.9 (5.9)	15.2 (3.3)
	Geriatric Depression Scale (GDS)	
	Apathy Evaluation Scale (self-rating)	
Seidl et al. (2011)	Erweitertes Autobiographisches Gedächtnis Inventar (E-AGI)	*N* = 239	Patients with MCI (*n* = 33)	21/12	79.3 (6.9)	
	Global Deterioration Scale (GDS)		Patients with mild AD (*n* = 35)	26/9	84.3 (7.8)	
	Mini mental state examination (MMSE)		Patients with moderate AD (*n* = 56)	49/7	86.9 (6.1)	
	Neuropsychiatric inventory (NPI)		Patients with severe AD (*n* = 74)	64/10	87.1 (7.0)	
	Apathy Evaluation Scale (AES-10)		Healthy controls (*n* = 41)	25/16	76.0 (4.7)	
Berna et al. (2012)	Erweitertes Autobiographisches Gedächtnis Inventar (E-AGI)	*N* = 395	MCI (*n* = 63)	29/34	74.0 (0.9)	12.3 (2.1)
	Nürnberger-Alters-Inventar (NAI)		Younger healthy controls (*n* = 194)	90/104	55.1 (1.0)	14.6 (2.5)
	Logical memory subtest (WMS-R)		Older healthy controls (*n* = 138)	73/65	73.8 (0.9)	13.9 (3.0)
	Trail Making Test, Versions A and B (TMT A, TMT B)	
Thomann et al. (2012)	Erweitertes Autobiographisches Gedächtnis Inventar (E-AGI)	*N* = 53	MCI (*n* = 15)	8/7	73.3 (3.8)	12.3 (3.1)
	Mini mental state examination (MMSE)		Mild AD (*n* = 14)	7/7	73.7 (5.2)	11.4 (3.0)
	Magnetic resonance imaging (MRI)		Healthy controls (*n* = 24)	10/14	72.8 (3.3)	13.8 (3.6)
Herold et al. (2013)	Erweitertes Autobiographisches Gedächtnis Inventar (E-AGI)	*N* = 54	Schizophrenia (*n* = 33)	10/23	52.0 (8.8)	12.6 (2.8)
	Brief Psychiatric Rating Scale (BPRS)		Healthy controls (*n* = 21)	9/12	53.7 (8.0)	13.9 (2.1)
	Scale for the Assessment of Positive Symptoms (SAPS)	
	Scale for the Assessment of Negative Symptoms (SANS)	
	Apathy Evaluation Scale (AES)	
	Bielefelder Famous Faces Test (BFFT)	
	Magnetic resonance imaging (MRI)	

**Figure 1:**
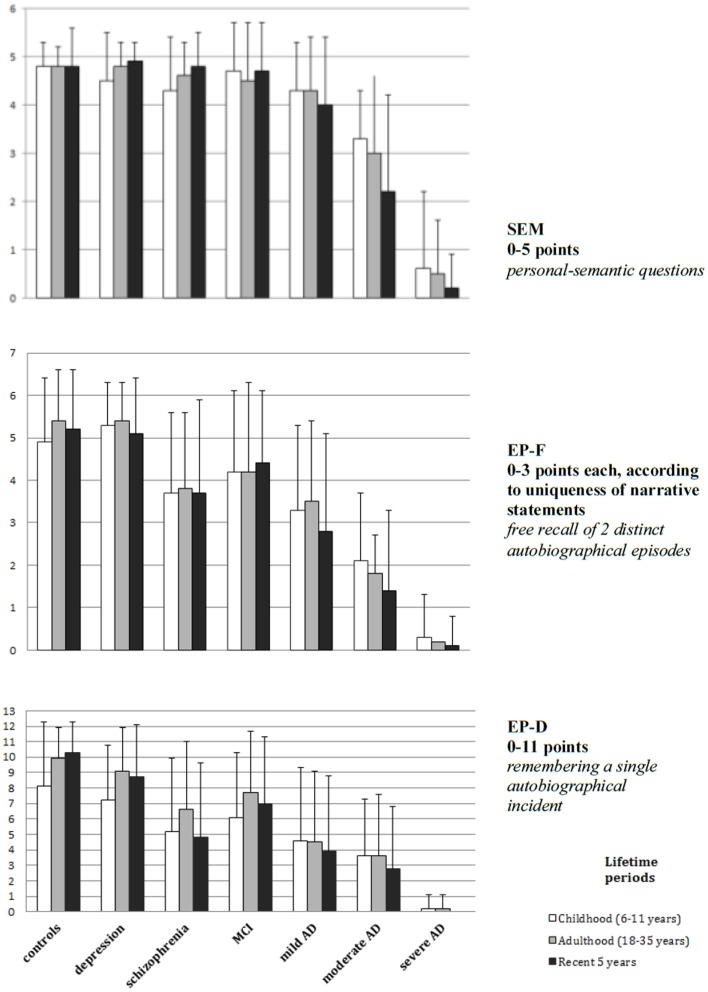
**Group comparison concerning semantic knowledge (SEM), free recalled episodes (EP-F) and episodic details (EP-D) by groups of patients with depression (Ahlsdorf, 2009), schizophrenia (Herold et al., 2013), MCI, different stages of AD and healthy controls (Seidl et al., 2011)**.

Autobiographical memory was investigated by using the Erweitertes Autobiographisches Gedächtnis Inventar (E-AGI) (Kopelman et al., 1990; Fast et al., 2007) – a semi-structured autobiographical interview based on the ABM Interview of Kopelman and colleagues. A previous version of the E-AGI was used in one study. Both, personal-semantic facts (SEM) as well as free recalled autobiographical events (EP-F) of five different lifetime periods (preschool, primary school, secondary school, early adulthood, recent 5 years) are considered. One autobiographical event from each lifetime period had to be described in detail. The score of maximal 11 points was given considering the number of details of such an event (EP-D). According to Conway (Conway, 1996; Conway and Pleydell-Pearce, 2000) event-specific knowledge plays a central role to autobiographical remembering and is stored and encoded in a completely different way than knowledge about “general events” or “lifetime periods,” which can be assigned to semantic autobiographical knowledge. To reduce the time necessary for the examination and to consider the restrictions due to the psychiatric conditions, the interview was modified and limited to the following three lifetime periods (primary school, early adulthood, recent 5 years – Figure 1) in four studies.

### Study 1: Psychometric properties of ABM assessment and effects of depressed mood (Ahlsdorf, 2009)

#### Group difference and effects of depressed mood

When compared between the four diagnostic groups, SEM scores showed only minor, non-significant differences. In contrast, EP-F scores were significantly higher in healthy controls, patients with major depression and patients with MCI than in those with manifest AD. Similar results applied for the EP-D which were significantly higher in the healthy controls followed by patients with MCI and major depression than in those with manifest AD. Only marginally, non-significant differences in EP-D scores between healthy controls and patients with major depression could be found. The E-AGI total values diminished non-significantly in patients with major depression in comparison to healthy controls. The study yielded an important result in the comparison of the evaluation of memories. Patients with major depression were occupied with negative thoughts and estimated their memories more negative than patients with AD.

### Study 2: ABM in nursing home residents with MCI and manifest AD (Seidl et al., 2011)

Autobiographical memory was examined in patients with different stages of AD and MCI, respectively, as well as in healthy controls (Table 1). Subjects were recruited in the framework of a large survey in nursing homes across Germany.

Results (Figure 1) demonstrated a progressive loss of ABM sum scores with increasing severity of dementia, which primarily involved episodic rather than semantic memories. When compared between controls, MCI, and mild AD diagnostic groups, SEM scores showed only minor, non-significant differences. Patients with moderate and severe AD displayed a significant reduction in SEM from the recent 5 years. Patients with moderate AD showed also a reduction for EP-F scores from the recent 5 years when compared to the childhood period whereas in healthy controls an inverse relationship was observed. This dissociation indicates that these memory functions are subserved by distinct neuronal systems as emphasized by the multiple trace hypothesis.

Further analyses of the temporal gradients in control subjects and MCI patients displayed a better memory performance from adulthood when compared to the childhood period. Both controls and patients with MCI showed lower EP-D scores for the childhood period.

In contrast, this recency effect was not found in patients with moderate AD suggesting an impact of the disease on the formation of recent memories.

### Study 3: ABM in normal aging and MCI (Berna et al., 2012)

Results confirmed a significant impairment of episodic ABM in MCI, but not in normal aging. Old-aged patients with MCI reached significantly lower scores than both Healthy Middle-Aged (*P* < 0.001) and Healthy Old-Aged (*P* = 0.02) subjects. Significant lower scores were also reached by Old-Aged patients with MCI compared with healthy Middle-Aged patients in the recent period (*P* = 0.004). Participants with MCI showed significantly lower scores than both control groups irrespective of age. These deficits were significantly correlated with verbal memory performances, but not with measures of executive functions.

### Study 4: Hippocampal changes and ABM in MCI and AD (Thomann et al., 2012)

Autobiographical memory deficits were investigated with respect to hippocampal changes in patients with MCI (*n* = 15), patients with mild AD and cognitively unaffected control subjects (*n* = 24) (Table 1). Associations between ABM sum scores and hippocampal changes were explored using partial correlations, each of the significant correlations was confirmed by regional shape analyses. Results confirmed a significant ABM loss in the in early stages of AD and in MCI. Episodic, but not semantic ABM losses were associated with hippocampal atrophy mainly involving the left hippocampus. Right-sided hippocampal atrophy corresponded to reduced scores in the EP-F of the “childhood” lifetime period. These associations referred to the regional rather than to the global hippocampal changes which primarily affect the hippocampal head and body.

### Study 5: ABM deficits in chronic schizophrenia (Herold et al., 2013)

Autobiographical memory BM and hippocampal volume were assessed in 33 patients with chronic schizophrenia (*n* = 24) or patients with schizoaffective disorder (*n* = 9) and 21 healthy volunteers matched for age, gender, and education. The assessment of ABM was part of a large neuropsychological test battery, which also addressed verbal, short-term, and working memory as well as remote semantic memory. Psychopathological symptoms were rated on appropriate rating scales (Table 1).

When compared with the healthy controls, patients showed a significantly poorer recollection of episodic ABM as well as a trend toward a lower performance with respect to semantic ABM. Analysis of MRI data revealed lower volumes of left anterior and posterior hippocampus as well as of the right posterior hippocampus in the patients group.

Both, episodic and semantic ABM-scores were significantly correlated with the left hippocampal volume in the patient group. This association applied for both, the left anterior as well as the left posterior part of the hippocampus. These associations accounted for 16% of the variance of episodic ABM and 24% of the variance of semantic ABM with educational level considered as a covariate.

## Discussion

The present studies yielded the following main findings: (i) a confirmation that episodic rather than semantic ABM is impaired in major psychiatric conditions such as AD and chronic schizophrenia; (ii) evidence that this effect is not accounted for by potential confounding factors such as age, education, or depressed mood; and (iii) an indication that ABM deficits refer to hippocampal changes in both AD and chronic schizophrenia.

That episodic rather than semantic ABM is impaired already in the early stages of AD including MCI is made evident by a wealth of studies. This effect involves the recognition of past events and also includes the remembrance of recent experiences such as a consultation in the doctors’ office and can facilitate clinical examination and diagnosis in early dementia (Donix et al., 2010). While semantic recall followed Ribot’s law in patients with manifest dementia in all stages, episodic ABM recall showed this effect in patients with mild and moderate dementia only, since the respective deficits also included earlier life time periods.

A significant effect of potential confounding variables – in particular age, education, or depressed mood – on these findings was not confirmed. Age is a variable difficult to consider in any study on AD since the disease progresses with it. We therefore investigated potential age effects in a 332 otherwise healthy volunteers from two birth cohorts and demonstrated only minor non-significant episodic ABM differences with age. School education had to be considered as another potential confounder since this variable is a robust marker of cognitive reserve (Fratiglioni and Wang, 2007; Sattler, 2011; Schröder and Pantel, 2011). However, an effect of school education could not be confirmed (Berna et al., 2012). Depressive mood was primarily considered by Ahlsdorf (2009) who described an effect on the emotional content of the memories reported rather than their recollection *per se*. Depressed patients showed a significantly higher rate of negative valuations in both, semantic and episodic ABM. Along with this, Seidl et al. (2011) did not find the severity of ABM deficits to be significantly correlated with depressive mood although their sample of 239 nursing home residents provided a sufficient effect size.

Two of the studies summarized here – each one involving patients with MCI and AD or patients with chronic schizophrenia – investigated ABM deficits with respect to MRI derived measures of hippocampal volume and shape. Irrespective of the diagnosis, episodic ABM deficits were associated with left hippocampal changes. An additional association of ABM deficits with right hippocampal changes was restricted to patients with MCI and AD. The respective associations clearly underline the importance of the hippocampus for the recollection of episodic ABM although these associations only accounted for a small proportion of the variance. Beginning in the early 1990s a wealth of neuroimaging studies found the hippocampus to be critically involved in MCI, AD, and chronic schizophrenia (Pantel et al., 1997; Heckers et al., 1998; Herold, 2011; Schröder and Pantel, 2011). Hence, it is plausible that the respective changes may result in similar deficits in both conditions. Differences refer to the extent of hippocampal changes and ABM deficits as well as to additional factors contributing to them. Further studies need to differentiate the association of hippocampal changes and ABM deficits by comparing hippocampal substructures for potential differences between these conditions or by considering additional clinical factors such as lifelong withdrawal, living without partnership, or long term hospitalization in patients with schizophrenia. Taken together, these finding conform with the multiple trace theory. Episodic ABM was already compromised in MCI and mild AD whereas recall of SEM was still preserved. This dissociation is generally referred to the hippocampus role for the recall of episodic but not semantic ABM since the former is already involved in the early and in the preclinical stages of AD (Pantel et al., 2003).

The results of our studies correspond to Conway’s formal differentiation of event-specific knowledge and “general events” or “lifetime periods” (Conway, 1996; Conway and Pleydell-Pearce, 2000). From a more phenomenological standpoint, the failure of episodic remembrance in the more advanced stages of AD and schizophrenia causes a breakdown of subjective coherence and identity since life stories (McAdams, 1985) stop to be accessible nor retrievable anymore. This effect may be associated with psychopathological symptoms such as apathy which is another common features in both AD and chronic schizophrenia.

In conclusion the present studies underline the importance of episodic ABM changes in MCI, AD, and chronic schizophrenia, i.e., conditions which share hippocampal changes as a common feature. While deficits of episodic ABM are already present in the early stages of AD, those of semantic ABM are confined to the more severe stages. In both, AD and chronic schizophrenia, ABM deficits were correlated with hippocampal changes. These findings demonstrate that ABM deficits can facilitate the clinical examination of patients with MCI, AD, and chronic schizophrenia.

## Conflict of Interest Statement

The authors declare that the research was conducted in the absence of any commercial or financial relationships that could be construed as a potential conflict of interest.
